# Valorization of Pumpkin Peel as a Source of Bioactive Compounds: Optimization of Heat- and Ultrasound-Assisted Extraction

**DOI:** 10.3390/molecules28073168

**Published:** 2023-04-02

**Authors:** Maria G. Leichtweis, Adriana K. Molina, Spyridon A. Petropoulos, Márcio Carocho, Tânia C. S. P. Pires, Maria Inês Dias, Ricardo Calhelha, M. Beatriz P. P. Oliveira, Carla Pereira, Lillian Barros

**Affiliations:** 1 Centro de Investigação de Montanha (CIMO), Instituto Politécnico de Bragança, Campus de Santa Apolónia, 5300-253 Bragança, Portugal; 2 Laboratório Associado para a Sustentabilidade e Tecnologia em Regiões de Montanha (SusTEC), Instituto Politécnico de Bragança, Campus de Santa Apolónia, 5300-253 Bragança, Portugal; 3 Department of Agriculture Crop Production and Rural Environment, University of Thessaly, 38446 Volos, Greece; 4 REQUIMTE—Science Chemical Department, Faculty of Pharmacy, University of Porto, Rua Jorge Viterbo Ferreira no. 228, 4050-313 Porto, Portugal

**Keywords:** *Cucurbita maxima* Duchesne, natural food preservatives, process optimization, phenolic compounds, pumpkin peel, bioactivity, RSM

## Abstract

The peels from three pumpkin genotypes cultivated in Greece were assessed for their phenolic content and bioactive properties to obtain extracts with a high preservative capacity. The optimization of the extraction was performed through response surface methodology (RSM) based on a Box–Behnken experimental design after applying two extraction techniques: heat-assisted (HAE) and ultrasound-assisted (UAE) extraction. The implemented independent variables were time, solvent concentration, and temperature/power (for HAE/UAE), while as dependent variables the dry residue (DR), reducing power (RP), and total phenolic content (TP) were considered. In general, HAE was the most effective technique for ‘TL’ (75 min; 30 °C; 24% ethanol) and ‘Voutirato’ (15 min; 30 °C; 10% ethanol), while UAE was more effective for ‘Leuka Melitis’ (5 min; 400 W; 0% ethanol). The extracts obtained in the global optimum conditions for each genotype peel were then assessed for their phenolic profile, by HPLC-DAD-ESI/MS, and bioactive potential. Seven phenolic compounds were detected, including four flavonoids, two phenolic acids, and one flavan-3-ol. The extracts presented high antioxidant, antibacterial, and antifungal potential, with no cytotoxicity for non-tumor cells. The optimized conditions for the extraction of preservative compounds from bioresidues were defined, allowing the acquisition of antioxidant and antimicrobial extracts and proving their potential for food application.

## 1. Introduction

The proper management of biowaste is essential in industrial processes, since its use can generate environmental and economic benefits and increase the added value in various industries. In particular, the by-products generated in the fruit and vegetable processing and juice and jam manufacturing industries, among others, represent a valuable source of bioactive compounds and could be used to obtain natural preservatives for food application. Studies have shown that the non-edible parts of fruits, such as the peel and seeds, contain high amounts of phenolic compounds [1,2]. For example, pumpkin peel is a rich source of these compounds and, beyond its antioxidant properties, it could also act as a natural preservative due to its high content of bioactive molecules. Recent research has shown that pumpkin peel extracts can inhibit the growth of bacteria and fungi, making them a natural alternative to chemical preservatives [3,4]. In fact, one study on the evaluation of the antimicrobial activity of pumpkin peel extract using the agar disc method demonstrated a remarkable broad-spectrum antimicrobial potential against the Gram-positive bacterium *Streptomyces viridochromogenes* [5], whereas Asif et al. reported the satisfactory antibacterial activity of pumpkin peel extracts against four bacterial strains (*Escherichia coli*, *Pasteurella multocida*, *Staphylococcus aureus*, and *Bacillus subtilis*) [6]. Moreover, pumpkin peels are rich in antioxidants such as ascorbic acid and tocopherols, which can contribute to the overall antioxidant capacity of this by-product [7], while other important bioactive compounds are carotenoids and β-carotene [8].

Numerous techniques have been investigated for the extraction, quantification, and identification of bioactive compounds, with the aim of discovering the optimal conditions for sustainable, cost-effective, and environmentally friendly processes. The key factors that can affect both the yield and bioactivity of these compounds are the extraction time, temperature, solvent type, and solid/liquid ratio. Therefore, it is critical to evaluate the impact of these factors to determine the ideal conditions that minimize losses and increase extraction yield [9]. Conventional methods such as heat-assisted extraction (HAE) expedite the transfer of compounds from the matrix to the solution and increase the solubility of bioactive compounds, generally resulting in higher extraction yields. However, these methods are limited because they may degrade bioactive compounds due to prolonged exposure to heat and result in the extraction of unwanted compounds, which can further impact the purity of the obtained extract. Thus, other methods such as ultrasound-assisted extraction (UAE) are being explored as an alternative to obtain these compounds. Ultrasound waves favor the release of compounds by breaking the matrix membranes through cavitation. This mechanism can reduce time and solvent consumption while increasing extraction yield and decreasing the likelihood of extracting unwanted compounds at the same time [10,11]. However, the comparison between two or more methods is necessary to verify the real effectiveness of each method on different matrices [12,13]. RSM is used to reduce the number of runs with different experimental conditions, thus reducing the time and money spent and maximizing the performance of the output. It uses statistical models to represent the relationship between the input variables and the outputs (the optimal conditions). It works by iteration, with each experiment (run) being used to refine the model and improve its predictions until an optimal solution (condition) is found. The Box–Behnken design is a type of RSM that uses a limited number of experimental runs to estimate a specific response. Its main advantages reside in the fact that it needs fewer runs than other designs as it uses evenly spaced levels for each variable. It includes a linear, quadratic, and interaction term. In this work, an RSM was implemented for both extraction techniques, although this did not constitute a literal comparison between the two due to the specific variables of each method [14]. The main objective was to detail the best conditions to obtain the highest antioxidant and antimicrobial activity for both extraction techniques.

Despite the abovementioned bioactivity of these bioresidues and the high volume generated by the food industry, to the best of our knowledge, few studies have been conducted on the extraction of bioactive compounds from pumpkin peels [3,15]. Therefore, this study was carried out with the aim of optimizing the extraction of preservative compounds from the peel of three pumpkin genotypes cultivated in Greece. For this purpose, a conventional technique (heat-assisted extraction) and an alternative technique (ultrasound-assisted extraction) were used, employing different extraction times, temperatures/power, and ethanol percentages. The process was designed by a response surface methodology (RSM), with the validation of the predictive models, in order to select those extracts that contained higher levels of total phenolic compounds while reducing the power requirements and solid residues. The extracts obtained through the optimized conditions were also assessed in terms of their phenolic compound profile, by HPLC-DAD-ESI/MS, and bioactivity, namely their antioxidant (cell-based assays), antimicrobial, and cytotoxic potential.

## 2. Results and Discussion

### 2.1. Optimized Responses and Conditions by RSM

The extraction of bioactive compounds from the peels of three different genotypes of pumpkin (P1, P2, and P3) was optimized in order to obtain preservative solutions. For this purpose, two different methods were compared: heat-assisted extraction (HAE) and ultrasound-assisted extraction (UAE). For each technique, an optimization procedure was performed through seventeen individual extractions, considering three independent variables. The response surface methodology (RSM) based on a Box–Behnken experimental design was applied. The independent variables, as well their ranges, were sourced from the literature [11]. The tested conditions determined by the Box–Behnken experimental design for time (*X*_1_ or t), temperature/power (*X*_2_ or T/P), and ethanol percentage (*X*_3_ or EtOH) are presented in Table 1.

The maximize function was chosen to obtain the highest values for dry residue (*R*_1_ or DR) and total phenols content (*R*_3_ or TP), while the minimize function was used for the reducing power (*R*_2_ or RP). Table 2 shows the results derived from the Box–Behnken experimental design used to optimize these dependent variables for each extraction technique. In some cases, not all the responses could be optimized, due to a significant lack of fit (α > 0.05). These results were analyzed using a quadratic Equation (12), applying the response surface methodology.

For the construction of the mathematical models, a confidence interval of α = 0.05 was used, excluding non-significant values. The final equations obtained to describe the evaluated responses using significant terms are presented in Table 3. The parametric values are presented as a function of the codification criteria of the experimental design, making it possible to compare the values between each other and interpret the weight of the influence of the numerical values on the responses.

Through a global analysis, it was possible to verify that the variable percentage of ethanol was more expressive in terms of both the linear effect and the quadratic effect, with a negative effect in most cases. As for the interactive effect, the interaction between ethanol and time, generally, had a higher effect than the interaction between time and temperature/power or ethanol and temperature/power.

To fulfill the objective of maximizing the extraction yield in terms of dry residue and total phenols, as well as obtaining extracts with a greater antioxidant capacity (minimizing the IC_50_ values, i.e., the sample concentration that provided 50% antioxidant activity), the values of the independent variables were adjusted to lead to an optimal response for each of the assessed extraction methods. It was possible to obtain optimal extraction conditions leading to optimal individual and global response values. These data are presented in Table 4 and subsequently discussed considering each pumpkin genotype. In addition, 3D surface graphs are presented for a better interpretation of the impact of the parametric values on the responses. In each figure, it is possible to observe the interaction between two of the three independent variables, keeping the third variable at a fixed level.

#### 2.1.1. Optimization of the Extraction for ‘TL’ Peel (P1)

For the *R*_1_ (DR) of sample P1, extracted through HAE, the analysis of the 17 runs rendered a quadratic function with a significant model, a non-significant lack of fit, an adjusted R^2^ of 0.984, and the coded Equation (1). Thus, the optimal values that maximized the amount of dry residue were set at 40 min, 70 °C, and 19% ethanol, which rendered 1.4 g/100g. The first row of Table 5 shows the 3D response charts for *R*_1_ at the optimal points. Overall, temperature and time did not have much influence on the optimization of the response, causing very low variation, while concentrations of ethanol beyond 40% seemed to reduce the dry residue yield.

Regarding *R*_2_ (RP), a quadratic function was obtained, although two runs were eliminated for being outliers, which allowed a non-significant lack of fit and an adequate fit of the model, with an adjusted R^2^ of 0.997, described by Equation (2). For this response, the minimize function was chosen, thus obtaining optimal values of 30 min, 31 °C, and 29% ethanol, rendering an estimated IC_50_ of 27 µg/g. The RSM charts can be found in the second row of Table 5, with the lowest values colored in dark blue. The two most important factors were extraction time and solvent percentage.

Finally, the *R*_3_ (TP) rendered a quadratic function with an adjusted R^2^ of 0.7203 and the coded Equation (3). The point that maximized the concentration of total phenols was under the conditions of 62 min, 80 °C, and 97% ethanol, which rendered 165 mg/g. The optimal values are shown in the third row of Table 5. Overall, the most important factors were temperature and ethanol percentage, although higher values for temperature should be considered due to the estimation of higher yields at temperatures over 80 °C.

The final row of Table 5 shows the charts of the desirability function, in which all three responses were considered, allowing for the determination of the optimal point of all responses. For this point, the parameters were set at 75 min, 30 °C, and 24% ethanol, rendering a DR of 1.28 g/100 g, an IC_50_ value of 158 µg/g for RP, and 136 mg/g of TP. This function allowed for an equilibrium of each response that could be individually reduced, but considering all the responses together, the conditions would be suitable and optimized for all.

For the UAE technique applied to this genotype, it was only possible to optimize *R*_1_. This response is detailed in Equation (4) with an adjusted R^2^ of 0.899. The optimal values for *R*_1_ were set at 400 W, 18 min, and 16% ethanol, which were expected to render 1.6 g/100g of dry residue. The 3D charts are shown on Table 6, in which it is clear that ethanol content showed a higher influence on the yield, while the ultrasonic power had only a slight influence.

#### 2.1.2. Optimization of the Extraction for ‘Voutirato’ Peel (P2)

For the *R*_1_ of sample P2, extracted through HAE, the analysis of the 17 runs rendered the quadratic Equation (5), with a significant model, a non-significant lack of fit, and an adjusted R^2^ of 0.975, although one run had to be eliminated for being an outlier. Thus, the optimal values that maximized the amount of dry residue were set at 120 min, 73 °C, and 24% ethanol, which rendered 1.515 g/100g. The first row of Table 7 shows the 3D response charts at the optimal values, in which it is possible to see that the temperature and extraction time had a weak effect on the variation of dry residue, and, once again, the ethanol content was the determinant, as for sample P1.

Regarding *R*_2_, the model chosen was a two-factor interaction, and the adjusted R^2^ of 0.7900 and the non-significant lack of fit for the 16 runs (one run did not show any antioxidant activity) are shown in the coded Equation (6). The minimize function resulted in an optimal point of 15 min, 30 °C, and only 2% ethanol, with an estimated IC_50_ value of 100 µg/g. The second row of Table 7 shows the 3D charts for this response. Considering the coded equation, the factor with the highest influence was temperature, followed by time, revealing that lower IC_50_ values were favored by lower temperatures and shorter extraction times.

An adequate modeling was not possible for the *R*_3_ of sample P2; thus, the third row of Table 7 represents the desirability of *R*_1_ and *R*_2_, which rendered an estimated 1.4 g/100 g of dry residue and 112 µg/g of reducing power at 15 min, 30 °C, and 10% ethanol.

As for the P1 variety, in the extraction of P2 by UAE, it was only possible to optimize *R*_1_. *R*_2_ analysis only rendered a linear model, and thus it was not considered for optimization studies, while *R*_3_ did not produce satisfactory results due to a lack of fit. In this technique, *R*_1_ rendered a quadratic function with a significant model, a non-significant lack of fit, and an adjusted R^2^ of 0.8748, described by the coded Equation (7). The model placed the optimal points at 380 W of power, 7 min, and 17% ethanol, while predicting 1.9 g/100 g of dry residue. Table 8 shows the 3D charts for sample P2, revealing that, to some extent, all the parameters influenced the optimal conditions for dry residue. A higher power, lower ethanol content, and shorter extraction time seemed to improve the dry residue quantity.

#### 2.1.3. Optimization of the Extraction for ‘Leuka Melitis’ Peel (P3)

For the *R*_1_ of sample P3 extracted by HAE, the analysis of the 17 runs rendered a quadratic function with a significant model, a non-significant lack of fit, and an adjusted R^2^ of 0.9660, as shown in the coded Equation (8). Thus, the optimal values that maximized the amount of dry residue were set at 98 min, 79 °C, and 27% ethanol, which were predicted to render 1.06 g/100 g of dry residue. In Table 9, the first row shows the 3D charts of *R*_1_ for this sample, indicating that, as in the previous samples, the temperature and time had a low influence on improving the yield, with the concentration of ethanol being the factor that markedly affected this response. The values obtained for the reducing power (*R*_2_) did not allow for a satisfactory model, so it was not included.

Considering *R*_3_, the values allowed for a significant model and non-significant lack of fit after removing one outlier. The coded equation is presented in Equation (9), and the adjusted R^2^ was 0.9161. For this response, the factor with the highest influence was also the percentage of ethanol, which can be seen graphically in the second row of Table 9. The optimal values after applying the maximize function were set at 68 min, 30 °C, and 100% ethanol, which rendered 135 mg/g of total phenols. Considering the desirability function for these two responses, the optimal values were set at 67 min, 30 °C, and 0% ethanol, for which 0.9 g/100 g of dry residue and 106 mg/g of total phenols were expected. The 3D charts of the desirability function are presented in the third row of Table 9.

Regarding the extraction of P3 by UAE, the analysis of the 17 runs rendered for *R*_1_ a quadratic function with a significant model, a non-significant lack of fit, and an adjusted R^2^ of 0.8970, as shown in the coded Equation (10). Through the interpretation of the coded equation, the factor with the highest influence seemed to be the power of the ultrasonic waves, as shown by the high values of the optimal point, which were set at 395 W of ultrasonic power, 9 min, and 31% ethanol, producing 1.25 g/100 g of dry residue. The 3D charts, displayed in the first row of Table 10, show a similar trend, with higher power intensities rendering higher yields of dry residue, while lower extraction times also seemed to favor this response. As with HAE, the *R*_2_ values did not allow for an optimization procedure.

Regarding *R*_3_, the values allowed for a quadratic model with a natural log transformation after ignoring two outliers. The model showed a significant fit and non-significant lack with an adjusted R^2^ of 0.9870, as shown in Equation (11). In the case of *R*_3_, the factor with the highest influence was the percentage of ethanol, as indicated by the coded values of the equation. The optimal values were set at 100 W, 29 min, and 100% ethanol, which were expected to yield 307 mg/g of total phenols, beyond what was achieved in the variation intervals of the factors. The second row of Table 10 shows the 3D charts for this response, highlighting the lower ultrasonic power requirements to promote higher total phenols, while high yields of ethanol and a longer extraction time seemed to promote the extraction yield of these bioactive molecules. The desirability function pointed towards optimum values of 80% ultrasound power, a 5 min extraction time, and 0% ethanol, which would render 1.12 g/100 g of dry residue and 120 mg/g of total phenols. The corresponding 3D charts are shown in the final row of Table 10.

#### 2.1.4. General Considerations

Overall, the HAE method seemed to be the best candidate for this optimization study, revealing the most robust results. It was clear that the concentration of ethanol had a higher influence on the dry residue yields for HAE, while temperature and time also showed moderate effects on the IC_50_ results for RP. Considering TP, the influence on this response was case-specific. According to the literature, a significant correlation was also recorded between the extraction conditions and the total phenolic compounds content and antioxidant activity in the case of *Berberis asiatica* fruit, which indicated the importance of optimizing the extraction protocol in order to achieve the highest extraction efficiency for bioactive compounds [16].

Regarding the UAE protocol, obtaining satisfactory values for the optimization was difficult, although the impact of the ethanol percentage on the dry yield variable was also determinant. Similarly to our study, Chen et al. [17] also suggested the importance of optimizing the solvent content in order to obtain the highest amounts of total phenolic compounds from *Lycium ruthenicum* Murr. (LR) fruit. Moreover, Asif et al. [6] suggested that the methanol content in the extraction solvent may also affect the antioxidant activity of squash peels, while different solvents may result in varied results in terms of the antioxidant capacity of squash fruit parts [18].

Considering all of the above, it was logical to select the HAE method as the most appropriate for obtaining extracts with a high residue content and antioxidant potential from pumpkin peels of genotypes 1 and 2, whereas for genotype 3, UAE led to better results. Although the UAE method for P3 demanded the highest power value tested (400 W), it resulted in a fast extraction time of only 5 min, which was the shortest time recorded, and required only water as solvent (0% ethanol). Low requirements in terms of extraction time (15 min) and ethanol content (10%) were also achieved by HAE for P2, with the advantage of also using the lowest temperature tested (30 °C). This low temperature was also the best for P1. According to the literature, the recovery of polyphenols from various bioresidues is highly dependent on the extraction protocol, with several studies suggesting that the ultrasound-assisted method is the most efficient compared to the solid-to-liquid extraction and microwave-assisted extraction protocols [19].

Despite the fact that some optimal values were found at the limits of the tested variables, it was unfeasible to try to overcome them. However, it is possible to affirm that the optimization process led to an efficient extraction protocol, saving both time and energy.

Many studies on the optimization of the extraction of bioactive compounds from pumpkins can be found in the literature [20,21,22,23] However, the use of pumpkin by-products as a source of such compounds and their use as food preservatives has still been scarcely explored. This work is an important contribution, being, to the best of our knowledge, the first study to optimize preservative compound extraction from pumpkin peels. Motivated by a real need within the pumpkin processing industry, these promising results encourage the replacement of synthetic preservatives, known to be related to adverse health effects, with a natural alternative obtained through sustainable processes. Furthermore, the evaluation of different pumpkin varieties, as well as the comparison of two extraction methodologies, made these results feasible and highly promising for upscaling. Through the predictive mathematical models obtained, it is also possible to simulate the best extraction conditions considering limitations of time, energy, and ethanol use.

### 2.2. Evaluation of the Extracts Obtained at the Optimum Conditions

In order to validate the predictive mathematical model of the studied process, experimental validation was performed with the estimated optimal extraction conditions, through which it was possible to verify the consistency within the predicted and real results obtained for the three responses considered in the optimization study (DR, RP, and TP; data not shown). Moreover, the optimal extracts were assessed for their phenolic compound profiles and antioxidant, antimicrobial, and cytotoxic properties in order to validate their potential for application as food preservatives.

#### 2.2.1. Phenolic Compound Profiles

**Figure 1:**
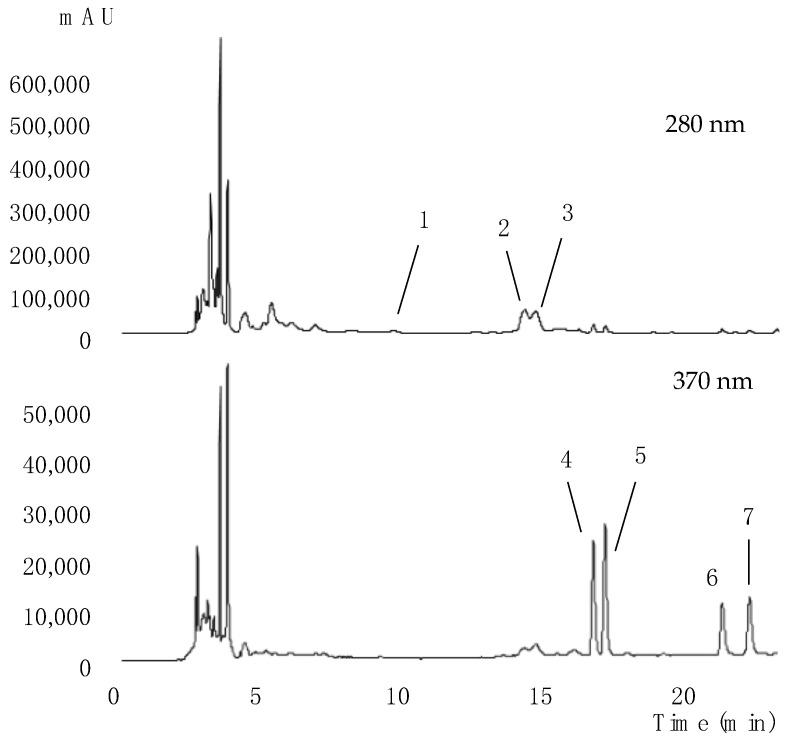
Chromatographic representation of the phenolic compounds profile obtained by HPLC-DAD under the optimal conditions for each extract.

The pumpkin peels were subjected to extraction under the global optimal conditions by HAE for P1 and P2, and by UAE for P3. These extracts were then evaluated by HPLC-DAD-ESI/MS to obtain their phenolic compound profiles. Table 11 presents the information of the UV-Vis at the maximum absorption, the deprotonated ion, the mass fragmentation, and the respective tentative identification of the compounds found in the extracts. The extracts from P1 and P2 showed the same profile with seven compounds detected, as presented in Figure 1, while sample P3 did not contain peak 6.

Peaks 1, 4, 5, 6, and 7 were tentatively identified considering the previous characterization of the extracts obtained from Portuguese and Algerian pumpkin peels [3]. (-)-Epicatechin ([M-H]^−^ at *m*/*z* 289, peak 1) was tentatively identified by comparison with the available standard, while the flavonoid compounds (peaks 4, 5, 6, and 7) were compared with the literature [24]. Regarding these flavonoids, peaks 4 ([M-H]^−^ at *m*/*z* 739) and 6 ([M-H]^−^ at *m*/*z* 593) presented only one MS^2^ fragment at *m*/*z* 285 (kaempherol aglycone), while peaks 5 ([M-H]^−^ at *m*/*z* 769) and 7 ([M-H]^−^ at *m*/*z* 623) presented the only MS^2^ fragment at *m*/*z* 315 (isorhamnetin aglycone). Thus, peaks 6 and 7, corresponded to the loss of two sugar moieties: deoxyhexosyl and hexoside ([M-H-146–162]^−^), which were tentatively identified as kaempferol-*O*-deoxyhexosyl-hexoside and isorhamnetin-*O*-deoxyhexosyl-hexoside, respectively. On the other hand, peaks 4 and 5, corresponded to three sugar moieties linked to the flavonoid aglycone ([M-H-146-146-162]^−^), which were tentatively identified as kaempferol-*O*-dideoxyhexosyl-hexoside and isorhamnetin-*O*-dideoxyhexosyl-hexoside, respectively.

In addition to the flavan-3-ol (peak 1) and the abovementioned flavonoids, the extracts also presented two phenolic acids (peaks 2 and 3). These peaks were tentatively identified as the *cis* and *trans* isomers of 4-*O*-*p*-coumaroylquinic acid, also called 4-pCoQA, presenting the pseudomolecular ion [M-H]^−^ at *m*/*z* 337 and the fragment ion MS^2^ as the base peak at *m*/*z* 173; 5% of the base peak at *m*/*z* 191 and *m*/*z* 135; and a maximum UV-Vis absorbance at 324/325 nm. These chromatographic responses were in accordance with those previously described in the literature [25], which have also been reported in African pumpkin leaves [26].

As shown in Table 12, for the three samples, the flavonoids group was the most abundant, with a total concentration of 2.05548 ± 0.00001 mg/g for P1, 1.7384 ± 0.0004 mg/g for P2, and 1.3388 ± 0.0002 mg/g for P3; however, for P2, the major compound was *cis*-4-*O*-*p*-coumaroylquinic acid (peak 2).

(-)-Epicatechin (peak 1) was the least representative compound in samples P1 and P2, contrarily to the results reported for the Portuguese pumpkin extracts, in which this was the most abundant compound [3]. The extracts from P1 and P2 showed higher contents of total phenolic compounds, approximately 3 mg/g each, while P3 presented almost half of the total concentration found in P1. These differences between the tested genotypes were in agreement with the report of Kiat et al. [27], who also recorded a variable content of polyphenols in the seeds of different pumpkin genotypes. Considering that in our study all the plants were grown under the same conditions, the effect of cultivation practices could be eliminated, and the genotypic effect could be suggested as the determinant factor.

#### 2.2.2. Antioxidant Activity

The optimized extracts were assessed by two biological assays, TBARS and OxHLIA, using homogenates of porcine brain and sheep erythrocytes, respectively, as oxidizable targets. These methods are considered more representative than colorimetric assays as they are cell-based and, thus, more similar to the reactions that occur in biological systems. The results obtained in both assays are shown in Table 13.

It is possible to notice the strong antioxidant capacity of the optimized extracts, especially for sample P1, which presented IC_50_ values of 850 ± 40 µg/mL for TBARS and 61 ± 1 µg/mL for OxHLIA, in agreement with its higher phenolic compounds content. The P2 extract provided an anti-hemolytic activity as strong as that presented by P1 and a lipid peroxidation inhibition capacity of 1600 µg/mL. P3 was the sample presenting the lowest antioxidant potential, in accordance with its lower phenolic compounds content. The IC_50_ values obtained in the present study were comparable or better than those reported for Portuguese and Algerian pumpkins [3], which presented IC_50_ values ranging from 88 to 209 µg/mL and 335 to 588 µg/mL for the OxHLIA assay and from 3921 to 7765 µg/mL and 2123 and 4569 µg/mL for the TBARS assay, respectively. These findings indicate the high antioxidant capacity of pumpkin fruit waste and highlight the potential of its further valorization in the food industry, since a significant correlation between the polyphenol extraction efficiency and the antiradical potential of the extracts has been well-confirmed [28].

#### 2.2.3. Antibacterial and Antifungal Activity

The activity presented by the optimum extracts tested against eight bacterial strains and two fungal strains is shown in Table 14. All the extracts were capable of inhibiting the growth of *Aspergillus brasiliensis*, *Listeria monocytogenes*, and *Staphylococcus aureus*. The P2 sample stood out and, in addition to these microorganisms, also presented bacteriostatic activity against the other five bacteria, whereas no samples were effective against *Bacillus cereus* at the maximum tested concentration (10 mg/mL). The best results were presented by the P2 sample against *Salmonella enterica* (MIC of 2.5 mg/mL) and by the P3 sample against *Yersinia enterocolitica* (MIC of 2.5 mg/mL). Moreover, P3 was also effective against *Pseudomonas aeruginosa* growth (MIC of 10 mg/mL) and P1 against *Enterobacter cloacae* and *Escherichia coli* (MIC of 10 and 5 mg/mL, respectively).

Research on the antimicrobial activity of pumpkin peels is very limited. In a study conducted in Pakistan in 2021, pumpkin peel, pulp, and seeds were tested and revealed significant antifungal activity against four strains: *Candida albicans*, *Fusarium oxysporum*, *Mucor miehei*, and *Trichoderma* spp. [29]. The authors also found antibacterial activity against *Salmonella typhi*, *Escherichia coli*, *Bacillus subtilis*, and *Streptococcus aureus* [29]. In a similar study, the antimicrobial activity of the peel, seeds, and fibers of pumpkin genotypes from Portugal and Algeria were assessed, and all Portuguese pumpkin samples demonstrated inhibitory activity against *Y. enterocolitica*, while those from Algeria inhibited *S. aureus*. In both studies, all samples inhibited the growth of *A. brasiliensis*, as in the present study [3].

#### 2.2.4. Cytotoxicity

The optimized extracts were assessed regarding their cytotoxicity in a primary culture of porcine liver cells (PLP2). None of the extracts presented cytotoxicity up to 400 µg/mL, which was an important first step in the verification of their safety when considering their use in the food industry. To the best of our knowledge, no other studies have reported potential toxic effects of pumpkin extracts.

## 3. Materials and Methods

### 3.1. Sample Preparation

During the 2020 growing season, three pumpkin genotypes were cultivated at the experimental field of the University of Thessaly in Velestino, central Greece (22.756E, 39.396 N), namely ‘Landrace from the region of Trikala’ (TL) (P1), ‘Voutirato’ (P2), and ‘Leuka Melitis’ (round) (P3). Fruits were harvested on October 2020, and the peels from 15 fruits from each genotype were separated; subjected to a freeze-drying process using a Sublimator model EKS, manufactured by Christian Zirbus Co. in Osterode am Harz, Germany; and then reduced to a fine powder (~20 mesh) by crushing for subsequent analysis.

### 3.2. Pumpkin Peel Extraction Procedures

#### 3.2.1. Heat-Assisted Extraction (HAE)

To perform the heat-assisted extraction of the powdered pumpkin peel samples, an internally stirred water reactor equipped with a CimarecTM magnetic stirrer (Thermo Scientific, San Jose, CA, USA) running at a constant speed of approximately 500 rpm was used. The extraction process followed a previously described procedure [30], whereby 0.5 g of sample was mixed with 20 mL of solvent, according to the conditions defined by the Box–Behnken experimental design as described in Table 1. The experimental parameters of the design were varied within the following ranges: time (t or *X*_1_) from 15 to 120 min, temperature (T or *X*_2_) from 30 to 80 °C, and ethanol content (EtOH or *X*_3_) from 0% (total water) to 100% (total ethanol). The solid-to-liquid ratio was kept constant at 25 g/L under all extraction conditions, based on previous optimization studies [31]. At the end of the extraction, the sample was filtered through Whatman No. 4 filter paper, and the supernatant was directly evaluated.

#### 3.2.2. Ultrasound-Assisted Extraction (UAE)

An ultrasonic device (QSonica sonicators, model CL-334, Newtown, CT, USA) equipped with a fixed water reactor at a frequency of 40 kHz was used for ultrasound-assisted extraction. The variables were programmed according to the Box–Behnken experimental design, as described in Table 1, following a previously described procedure [12]. Samples of pumpkin peels from each genotype (0.75 g) were placed in a reactor with 30 mL of solvent and were extracted under the planned conditions, keeping the solid/liquid ratio constant at 25 g/L. The experimental design ranges were set as follows: time (t or *X*_1_) from 5 to 60 min; ultrasonic power (P or *X*_2_) from 100 (20% of the total equipment power rating) to 400 W (80%); and ethanol ratio (EtOH or *X*_3_) from 0% to 100%. At the end of the extraction, the extract was filtered through Whatman No. 4 filter paper, and the supernatant was directly used for analysis.

### 3.3. Response Value Formats for Result Presentation

The optimized responses for dry residue (DR or *R*_1_), reducing power (RP or *R*_2_), and total phenolic content (TP or *R*_3_) were determined.

The DR (*R*_1_) was obtained by drying 3 mL of extract at 105 °C for 48 h and was expressed in g/100 g.

The RP (*R*_2_) was evaluated considering the ability of the extracts to reduce Fe^3+^. For this purpose, 0.5 mL of the extract, in serial dilutions using the respective extraction solvent, and the same volume of sodium phosphate buffer solution (0.2 M, pH 6.6) and potassium ferricyanide (1%) were mixed and incubated at 50 °C for 20 min; then, the reaction was stopped with 0.5 mL of trichloroacetic acid (10%). Next, 0.8 mL was mixed with 0.8 mL of distilled water and 160 μL of ferric chloride (0,1%) in a 48-well microplate, and the absorbance was measured at 690 nm [32]. The results were expressed as IC_50_ values, referring to the extract concentration necessary to inhibit the iron reduction by 50%, expressed in µg/mL.

Finally, the TP (*R*_3_) was assessed by the Folin–Ciocalteu (F–C) methodology, where 0.5 mL of extract was mixed with 2.5 mL of the F–C reagent (1:10 *v*/*v*) and 2 mL of sodium carbonate (75 g/L). After incubation in a water bath for 30 min at 40 °C, the absorbance was measured at 765 nm [32]. The results were expressed in gallic acid equivalents, in milligrams per gram of extract (mg/g).

The objective function used for DR and TP was “maximize” to obtain the highest possible combination of factors, while for RP, the objective function used was “minimize”, since for IC_50_ values, lower concentrations represent better results.

### 3.4. Experimental Design, Model Analysis, and Statistical Evaluation

The study was conducted using an independent quadratic Box–Behnken design (BBD). The BBD is a three-level-three-factor system, through which it was possible to determine the best combination of extraction variables that led to extracts with greater responses (DR, RP, and TP) [33]. The experimental design, as well as the response values, are recorded in Table 1. The predictive mathematical models were derived from the quadratic Equation (12), in which R_n_ are the responses and b_n_ are the interception, linear, quadratic, and interaction terms.

R_n_ = b_0_ + b_1_*X*_1_ + b_2_*X*_2_ + b_3_*X*_3_ + b_12_*X*_1_*X*_2_ + b_13_*X*_1_*X*_3_ + b_23_*X*_2_*X*_3_ + b_11_*X*_1_^2^ + b_22_*X*_2_^2^ + b_33_*X*_3_^2^ (12)

The coefficient of determination (R^2^) of the model equations was obtained using analysis of variance (ANOVA), and the significances (α = 0.05) were verified by an *F*-test. Design Expert software 8.0.6 (State-Ease Inc., Minneapolis, MN, USA) was used for the experimental design and analysis.

### 3.5. Phenolic Compounds Analysis by HPLC-DAD-ESI/MS

The pumpkin peel extracts were dissolved in an ethanol–water solution (20:80, *v*/*v*) to obtain solutions with a concentration of 10 mg/mL and then filtered through a 0.22 μm nylon syringe filter. The phenolic compounds profile was determined by HPLC coupled to a diode array detector and electrospray ionization-mass spectrometry (HPLC-DAD-ESI/MS) [34]. The tentative identification of the compounds was based on the obtained information, including retention times and UV-Vis and mass spectra, as well as through comparison with commercial standards and available bibliographic information. Quantification was carried out by measuring the area of the peaks obtained and comparing them with the calibration curves of the most similar commercially available standards. The final results were expressed in mg/g of extract.

### 3.6. Evaluation of Bioactive Properties

The antioxidant, antimicrobial, and cytotoxic activity of the three extracts obtained under optimal extraction conditions was evaluated.

#### 3.6.1. Antioxidant Activity

The ability to inhibit lipid peroxidation was assessed via the thiobarbituric acid reactive substances inhibition (TBARS) assay, using porcine (*Sus scrofa*) brain homogenates. The antioxidant potential was measured by observing the reduction in the level of TBARS, as outlined by Pereira et al. [35]. The IC_50_ value, which represents the sample concentration providing 50% antioxidant activity, was used to express the results in μg/mL. In addition, the anti-hemolytic activity of the extracts was evaluated using the oxidative hemolysis inhibition assay (OxHLIA), described by Lockowandt et al. [36], and the results were expressed in μg/mL as the IC_50_ value. This value indicated the concentration of the sample that could cause a delay of 60 min in oxidative hemolysis. To serve as a positive control, Trolox was used in both assays.

#### 3.6.2. Antimicrobial Activity

To determine the antimicrobial potential of the samples, a variety of microorganisms were examined, including different types of bacteria and fungi. The bacterial strains comprised both Gram-positive (*Bacillus cereus* (ATCC 11778), *Staphylococcus aureus* (ATCC 25923), and *Listeria monocytogenes* (ATCC 19111)) and Gram-negative strains (*Escherichia coli* (ATCC 25922), *Enterobacter cloacae* (ATCC 49741), *Pseudomonas aeruginosa* (ATCC 9027), *Salmonella enterica* subsp (ATCC 13076), and *Yersinia enterocolitica* (ATCC 8610)). The analyzed fungi were *Aspergillus fumigatus* (ATCC 204305) and *Aspergillus brasiliensis* (ATCC 16404). The antibacterial and antifungal activity were determined using the method outlined by Heleno et al. [37]. Both the minimum inhibitory concentration (MIC) and the minimum bactericidal (MBC) or minimum fungicidal (MFC) concentrations were determined for the bacteria and fungi. The positive controls used for the bacteria were streptomycin and ampicillin, and for the fungi, ketoconazole and bifonazole. The results were expressed in mg/mL.

#### 3.6.3. Cytotoxic Activity

In order to assess the cytotoxicity, a primary culture of non-tumorigenic porcine liver cells (PLP2) was used, obtained from freshly harvested porcine liver purchased from a local slaughterhouse. The sulforhodamine B (SRB) colorimetric assay was carried out with ellipticine as a positive control [38]. The IC_50_ values (the concentration of extract inhibiting 50% of net cell growth) were expressed in μg/mL.

### 3.7. Statistical Analysis

The samples were analyzed in triplicate, and the results were expressed as mean ± standard deviation. For only two groups of data, a Student’s t-test was used for comparison, and one-way analysis of variance (ANOVA) was used for three or more groups. The normal distribution and homogeneity of variance were evaluated using Shapiro–Wilk and Levene tests, respectively. Homoscedastic data with *p* > 0.05 were analyzed using a Tukey’s honestly significant difference (HSD) test. All tests were conducted at a significance level of 5% using IBM SPSS Statistics software (Version 22.0, IBM Corp, Armonk, NY, USA).

## 4. Conclusions

Two extraction methodologies, heat- (HAE) and ultrasound-assisted extraction (UAE), were compared through an RSM optimization study to obtain pumpkin peel extracts rich in food preservatives. The HAE methodology proved to be more efficient for the ’TL’ genotype, making it possible to reduce the extraction temperature (to 30 °C) and using low concentrations of ethanol in water (24%). For the ‘Voutirato’ genotype, apart from requiring low temperatures and ethanol concentrations (10%), this technology (HAE) also allowed a low extraction time (15 min). On the other hand, for the ‘Leuka Melitis’ genotype, UAE proved to be more efficient, making it possible to save time (5 min of extraction time) and use only water (0% ethanol) as the extraction solvent. The optimization by RSM allowed us to find the conditions that led to a higher extraction yield, reducing power, and total phenols concentration with reduced time, energy, and solvent parameters. The use of the Box–Behnken experimental design allowed the simultaneous evaluation of three independent variables in a wide range through 17 experimental runs.

The extracts obtained under the global optimal conditions were characterized regarding their phenolic profiles by HPLC-DAD-ESI/MS, where the ’TL’ and ‘Voutirato’ genotypes presented the highest contents of phenolic compounds (3.026 ± 0.008 and 2.824 ± 0.001 mg/g, respectively). Moreover, the obtained optimal extracts demonstrated a high antioxidant potential in the TBARS and OxHLIA assays and the capacity to inhibit the growth of at least four of the eight bacterial strains tested and the fungus *Aspergillus brasiliensis* without revealing cytotoxicity in non-tumor liver cells. These bioactive properties corroborated the potential of the obtained extracts to act as food preservatives, while the optimization of the extraction protocols may allow an improvement in extraction efficiency and increase the added value of the crop through the valorization of peel waste for food preservation purposes.

## Figures and Tables

**Table 1:** Time, temperature (for the heat-assisted extraction; HAE) or power (for the ultrasound-assisted extraction; UAE), and percentage of ethanol (EtOH) in the solvent, obtained from Box–Behnken experimental design for the extraction optimization.

Run	HAE			UAE		
	*X*_1_ − t (min)	*X*_2_ − T (°C)	*X*_3_ − EtOH (%)	*X*_1_ − t (min)	*X*_2_ – P (% of W) ^1^	*X*_3_ − EtOH (%)
1	67.5	30	0	32.5	20	0
2	120	55	100	60	20	50
3	67.5	55	50	5	80	50
4	67.5	55	50	5	50	0
5	67.5	80	0	60	50	0
6	67.5	80	100	32.5	50	50
7	15	80	50	32.5	50	50
8	15	55	100	32.5	80	0
9	67.5	55	50	32.5	50	50
10	15	30	50	32.5	50	50
11	67.5	30	100	60	80	50
12	120	55	0	5	50	100
13	67.5	55	50	32.5	20	100
14	120	30	50	5	20	50
15	120	80	50	32.5	50	50
16	67.5	55	50	60	50	100
17	15	55	0	32.5	80	100

^1^ The ultrasonic power variable was designed considering the maximum power rating of the equipment (500 W) as 100%.

**Table 2:** Results of the extraction runs of the independent variables of time, temperature/power, and ethanol percentage in the solvent for the two extraction techniques applied (heat-assisted extraction: HAE; and ultrasound-assisted extraction: UAE) and for the three dependent variables evaluated (*R*_1_, g/100g; *R*_2_, µg/mL; and *R*_3_, mg/g).

Run	P1						P2						P3					
	HAE			UAE			HAE			UAE			HAE			UAE		
	*R*_1_	*R*_2_	*R*_3_	*R*_1_	*R*_2_	*R*_3_	*R*_1_	*R*_2_	*R*_3_	*R*_1_	*R*_2_	*R*_3_	*R*_1_	*R*_2_	*R*_3_	*R*_1_	*R*_2_	*R*_3_
	DR	RP	TP	DR	RP	TP	DR	RP	TP	DR	RP	TP	DR	RP	TP	DR	RP	TP
1	1.3	118	132	1.1	25	163	1.4	244	120	1.4	147	131	0.9	34	105	0.9	215	91
2	0.7	169	126	0.9	38	178	0.7	101	77	1.3	100	90	0.4	202	107	0.8	157	100
3	1.3	329	142	1.6	206	142	1.3	304	131	1.9	495	107	0.9	1333	85	1.2	423	70
4	1.3	313	112	1.2	319	170	1.4	405	116	1.4	337	132	0.9	664	80	1.0	703	111
5	1.3	401	98	1.2	304	127	1.9	612	91	0.6	295	86	1.0	1013	94	0.9	607	64
6	0.8	830	163	1.1	191	127	0.9	351	127	1.3	341	97	0.4	561	125	0.9	461	81
7	1.3	924	124	1.0	856	139	1.4	345	88	1.2	420	96	0.9	608	115	0.8	484	81
8	0.6	274	108	1.5	323	143	0.7	348	99	1.5	340	97	0.3	455	107	1.0	762	108
9	1.2	345	103	1.1	262	133	1.3	416	96	1.3	316	99	0.9	550	78	0.9	736	75
10	1.1	85	98	1.1	93	144	1.3	128	113	1.2	159	86	0.8	148	83	0.7	235	75
11	0.5	131	128	1.2	35	133	0.6	143	124	1.4	103	99	0.2	82	134	0.9	173	73
12	1.3	401	104	0.2	317	180	1.4	504	101	0.5	346	117	0.9	551	87	0.2	348	113
13	1.3	334	107	0.3	316	144	1.4	388	89	0.4	369	147	0.9	498	68	0.1	-	113
14	1.1	261	138	0.9	197	134	1.3	263	113	1.2	377	97	1.0	477	64	0.6	621	274
15	1.3	416	121	1.4	575	106	1.5	470	97	1.3	408	57	1.1	741	76	0.7	628	74
16	1.3	366	117	0.3	267	153	1.3	359	104	0.7	502	126	1.0	597	70	0.1	-	181
17	1.3	332	48	0.5	117	96	1.3	-	132	0.7	267	100	0.9	1066	65	0.7	1528	37

**Table 3:** Mathematical models derived from the second-order polynomial model with interaction described in Equation (12) in terms of encoded values for the two extraction techniques (heat-assisted extraction: HAE; and ultrasound-assisted extraction: UAE) and the three responses, when available (*R*_1_ or DR; *R*_2_ or RP; and *R*_3_ or TP).

P1	MAC			Equation
	Dry residue (DR)	*R*_1_ =	1.27 + 0.0714*X*_1_ + 0.0171*X*_2_ − 0.3232*X*_3_ + 0.0027*X*_1_*X*_2_ − 0.0666*X*_1_*X*_3_ + 0.0169*X*_2_*X*_3_ − 0.0396*X*_1_^2^ − 0.0245*X*_2_^2^ − 0.2882*X*_3_^2^	(1)
	Reducing power (RP)	*R*_2_ =	337.4 + 247*X*_1_ − 83*X*_2_ + 110.5*X*_3_ − 171*X*_1_*X*_2_ + 104*X*_1_*X*_3_ − 226.5*X*_2_*X*_3_ + 43.05*X*_1_^2^ + 41.05*X*_2_^2^ − 10.45*X*_3_^2^	(2)
	Total phenols (TP)	*R*_3_ =	111.24 + 1.21*X*_1_ + 13.861*X*_2_ + 17.97*X*_3_ + 10.83*X*_1_*X*_2_ + 17.25*X*_1_*X*_3_ + 9.69*X*_2_*X*_3_ + 18.86*X*_1_^2^ − 14.93*X*_2_^2^ − 4.85*X*_3_^2^	(3)
	UAE			
	Dry residue (DR)	*R*_1_ =	1.14 − 0.0537*X*_1_ − 0.2023*X*_2_ − 0.4659*X*_3_ − 0.0954*X*_1_*X*_2_ + 0.0077*X*_1_*X*_3_ − 0.0401*X*_2_*X*_3_ − 0.0343*X*_1_^2^ − 0.0716*X*_2_^2^ − 0.3612*X*_3_^2^	(4)
P2	MAC			
	Dry residue (DR)	*R*_1_ =	1.34 + 0.0675*X*_1_ + 0.0255*X*_2_ − 0.33147*X*_3_ + 0.0179*X*_1_*X*_2_ + 0.0811*X*_1_*X*_3_ − 0.0129*X*_2_*X*_3_ − 0.01273*X*_1_^2^ + 0.0232*X*_2_^2^ − 0.3211*X*_3_^2^	(5)
	Reducing power (RP)	*R*_2_ =	332.03 + 125*X*_1_ + 31.68*X*_2_ − 85.07*X*_3_ − 2.5*X*_1_*X*_2_ − 40*X*_1_*X*_3_ − 121.86*X*_2_*X*_3_	(6)
	UAE			
	Dry residue (DR)	*R*_1_ =	1.25 − 0.1139*X*_1_ − 0.1471*X*_2_ − 0.3450*X*_3_ − 0.1515*X*_1_*X*_2_ + 0.2518*X*_1_*X*_3_ − 0.0328*X*_2_*X*_3_ − 0.0114*X*_1_^2^ + 0.1861*X*_2_^2^ − 0.4336*X*_3_^2^	(7)
P3	MAC			
	Dry residue (DR)	*R*_1_ =	0.9179 + 0.0555*X*_1_ + 0.0473*X*_2_ − 0.3145*X*_3_ − 0.0138*X*_1_*X*_2_ + 0.0462*X*_1_*X*_3_ + 0.0145*X*_2_*X*_3_ − 0.0140*X*_1_^2^ + 0.0308*X*_2_^2^ − 0.3241*X*_3_^2^	(8)
	Total phenols (TP)	*R_3_* =	76.08 − 6.04*X*_1_ + 4.46*X*_2_ + 15.20*X*_3_ + 12.93*X*_1_*X*_2_ + 0.5*X*_1_*X*_3_ − 5.4*X*_2_*X*_3_ + 6.68*X*_1_^2^ − 16.33*X*_2_^2^ + 31.75*X*_3_^2^	(9)
	UAE			
	Dry residue (DR)	*R*_1_ =	0.7940 − 0.0405*X*_1_ + 0.1595*X*_2_ − 0.3463*X*_3_ − 0.1440*X*_1_*X*_2_ − 0.0025*X*_1_*X*_3_ + 0.1160*X*_2_*X*_3_ − 0.02*X*_1_^2^ + 0.1240*X*_2_^2^ − 0.2350*X*_3_^2^	(10)
	Total phenols (TP)	*R_3_* =	4.35 − 0.2536*X*_1_ − 0.4320*X*_2_ + 0.0063*X*_3_ + 0.2625*X*_1_*X*_2_ + 0.0095*X*_1_*X*_3_ − 0.5298*X*_2_*X*_3_ + 0.1029*X*_1_^2^ + 0.2490*X*_2_^2^ + 0.0042*X*_3_^2^	(11)

**Table 4:** Optimized conditions of the independent variables, presented as real values, with which the optimal values for the individual and global responses were obtained.

Sample	Criteria	Heat-Assisted Extraction (HAE)					Ultrasound-Assisted Extraction (UAE)				
		Optimal Variable Conditions			Optimum Response		Optimal Variable Conditions			Optimum Response	
		*X*_1_: t (min)	*X*_2_: T (°C)	*X*_3_: EtOH (%)			*X*_1_: t (min)	*X*_2_: P (W)	*X*_3_: EtOH (%)		
P1	*Individual optimal variable conditions*										
	*R*_1_	40	70	19	1.4	g/100 g dw	18	400	16	1.6	g/100 g dw
	*R*_2_	30	31	29	27	µg/mL	-	-	-	-	µg/mL
	*R*_3_	62	80	97	165	mg/g dw	-	-	-	-	mg/g dw
	*Global optimal variable conditions*										
	*R*_1_	75	30	24	1.3	g/100 g dw	-	-	-	-	g/100 g dw
	*R*_2_				158	µg/mL				-	µg/mL
	*R*_3_				136	mg/g dw				-	mg/g dw
P2	*Individual optimal variable conditions*										
	*R*_1_	120	73	24	1.5	g/100 g dw	7	380	17	1.9	g/100 g dw
	*R*_2_	15	30	2	100	µg/mL	-	-	-	-	µg/mL
	*R*_3_	-	-	-	-	mg/g dw	-	-	-	-	mg/g dw
	*Global optimal variable conditions*										
	*R*_1_	15	30	10	1.4	g/100 g dw	-	-	-	-	g/100 g dw
	*R*_2_				112	µg/mL				-	µg/mL
	*R*_3_				-	mg/g dw				-	mg/g dw
P3	*Individual optimal variable conditions*										
	*R*_1_	98	79	27	1.1	g/100 g dw	9	395	31	1.3	g/100 g dw
	*R*_2_	-	-	-	-	µg/mL	-	-	-	-	µg/mL
	*R*_3_	68	30	100	135	mg/g dw	>29	>100	>100	>307	>mg/g dw
	>*Global optimal variable conditions*										
	*R*_1_	67	30	0	0.9	g/100 g dw	5	400	0	1.1	g/100 g dw
	*R*_2_				-	µg/mL				-	µg/mL
	*R*_3_				106	mg/g dw				120	mg/g dw

**Table 5:** 3D response charts of the heat-assisted extraction (HAE) of ‘TL’ peel (P1) at the optimal values.

Sample P1 with HAE			
	Temperature vs. Time	Solvent vs. Time	Solvent vs. Temperature
*R*_1_ − DR	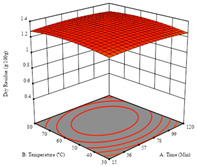	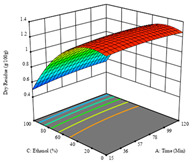	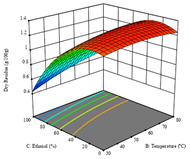
*R*_2_ − RP	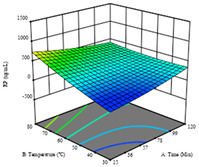	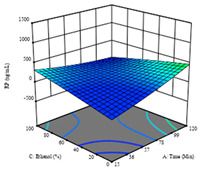	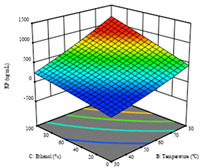
*R*_3_ − TP	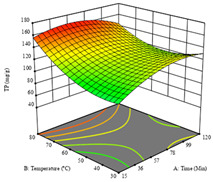	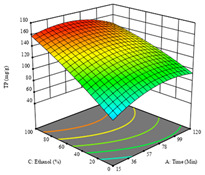	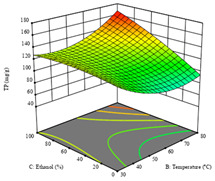
Desirability	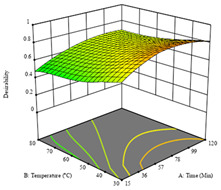	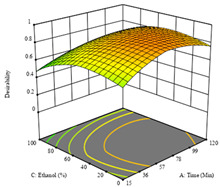	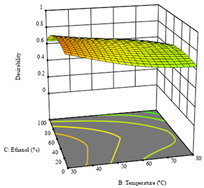

**Table 6:** 3D response charts of the ultrasound-assisted extraction (UAE) of ‘TL’ peel (P1) at the optimal values.

Sample P1 with UAE			
	Power vs. Time	Power vs. Solvent	Time vs. Solvent
*R*_1_ − DR	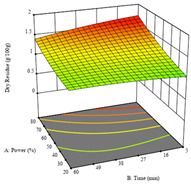	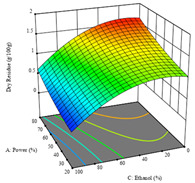	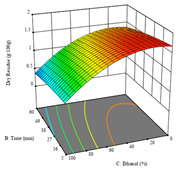

**Table 7:** 3D response charts of the heat-assisted extraction (HAE) of ‘Voutirato’ peel (P2) at the optimal values.

Sample P2 with HAE			
	Temp. vs. Time	Solvent vs. Time	Solvent vs. Temperature
*R*_1_ − DR	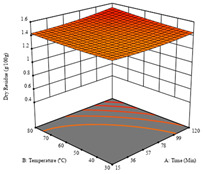	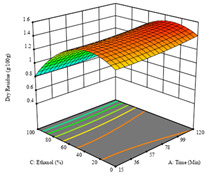	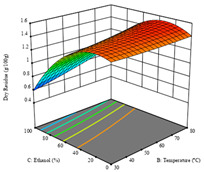
*R*_2_ − RP	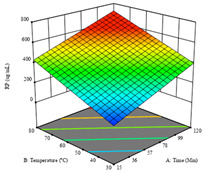	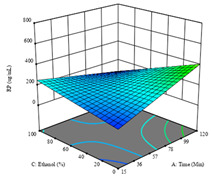	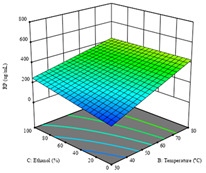
Desirability	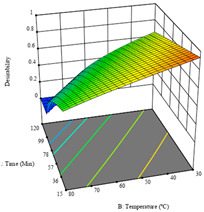	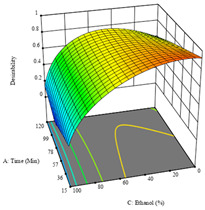	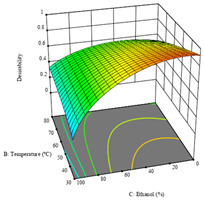

**Table 8:** 3D response charts of the ultrasound-assisted extraction (UAE) of ‘Voutirato’ peel (P2) at the optimal values.

Sample P2 with UAE			
	Power vs. Time	Solvent vs. Power	Solvent vs. Time
*R*_1_ − DR	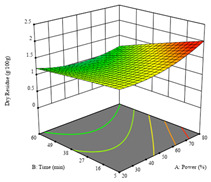	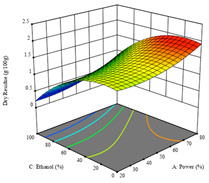	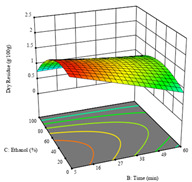

**Table 9:** 3D response charts of the heat-assisted extraction (HAE) of ‘Leuka melitis’ peel (P3) at the optimal values.

Sample P3 with HAE			
	Temp. vs. Time	Solvent vs. Time	Solvent vs. Temperature
*R*_1_ − DR	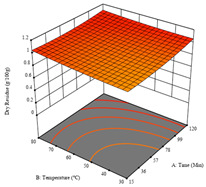	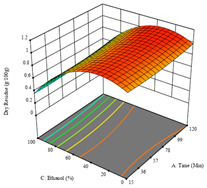	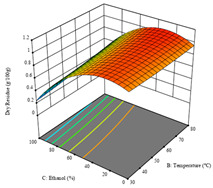
*R*_3_ − TP	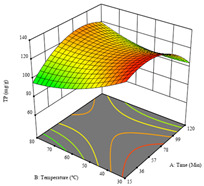	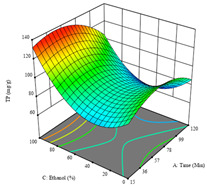	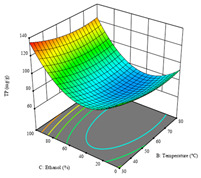
Desirability	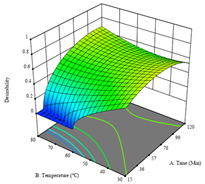	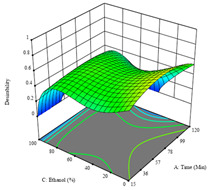	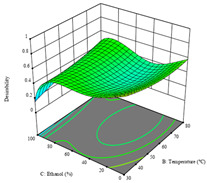

**Table 10:** 3D response charts of the UAE of ‘Leuka melitis’ peel (P3) at the optimal values.

Sample P3 with UAE			
	Power vs. Time	Solvent vs. Power	Time vs. Solvent
*R*_1_ − DR	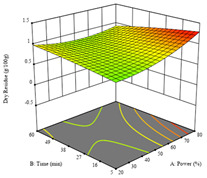	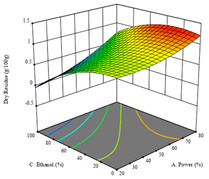	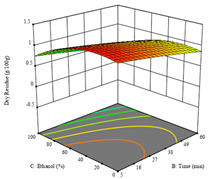
*R*_3_ − TP	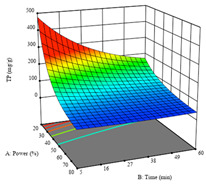	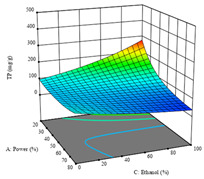	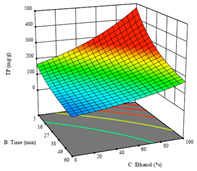
Desirability	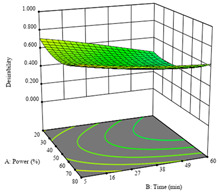	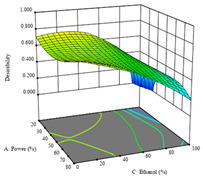	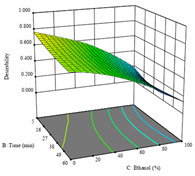

**Table 11:** Characterization of the phenolic compounds detected under the optimal conditions for each extract by HPLC-DAD-ESI/MS.

Peak	Rt (min)	λmax (nm)	[M-H]^−^ (*m*/*z*)	MS^2^ (*m*/*z*)	Tentative Identification
1	7.69	280	289	245(100),205(45)	(-)-Epicatechin
2	14.11	324	337	191(5),173(100),135(5)	*cis*-4-*O*-*p*-Coumaroylquinic acid
3	14.49	325	337	191(5),173(100),135(5)	*trans*-4-*O*-*p*-Coumaroylquinic acid
4	16.51	354	739	285(100)	Kaempferol-*O*-dideoxyhexosyl-hexoside
5	16.93	354	769	315(100)	Isorhamnetin-*O*-dideoxyhexosyl-hexoside
6	21.03	348	593	285(100)	Kaempferol-*O*-deoxyhexosyl-hexoside
7	21.98	365	623	315(100)	Isorhamnetin-*O*-deoxyhexosyl-hexoside

**Table 12:** Quantification of the phenolic compounds detected under the optimal conditions for each extract (mg/g of extract).

Peak	P1	P2	P3
1	0.244 ± 0.007 ^a^	0.210 ± 0.005 ^b^	0.091 ± 0.004 ^c^
2	0.355 ± 0.003 ^b^	0.52 ± 0.02 ^a^	0.0583 ± 0.0003 ^c^
3	0.371 ± 0.004 ^a^	0.36 ± 0.02 ^a^	0.0367 ± 0.0003 ^b^
4	0.5204 ± 0.0006 ^a^	0.42085 ± 0.00007 ^c^	0.444589 ± 0.000009 ^b^
5	0.5302 ± 0.0003 ^a^	0.4800 ± 0.0003 ^b^	0.45000 ± 0.00006 ^c^
6	0.5005 ± 0.0005 ^a^	0.41053 ± 0.00004 ^b^	n.d.
7	0.5044 ± 0.0002 ^a^	0.42703 ± 0.00009 ^c^	0.4442 ± 0.0002 ^b^
Total flavan-3-ols	0.244 ± 0.007 ^a^	0.210 ± 0.005 ^b^	0.091 ± 0.004 ^c^
Total phenolic acids	0.7261 ± 0.0006 ^b^	0.876 ± 0.005 ^a^	0.09495 ± 0.00009 ^c^
Total flavonoids	2.05548 ± 0.00001 ^a^	1.7384 ± 0.0004 ^b^	1.3388 ± 0.0002 ^c^
Total phenolic compounds	3.026 ± 0.008 ^a^	2.824 ± 0.001 ^b^	1.525 ± 0.004 ^c^

n.d.—not detected. Calibration curves used for quantification: (-)-catequin (*y* = 84,950*x* − 23,200, R^2^ = 0.999, LOD = 0.17 µg/mL, LOQ = 0.68 µg/mL, peak 1); *p*-coumaric acid (*y* = 301,950*x* + 6966.7, R^2^ = 0.9995, LOD = 0.71 µg/mL, LOQ = 2.38 µg/mL, peaks 2 and 3); and quercetin 3-*O*-glucoside (*y* = 34,843*x* − 160,173; R^2^ = 0.9998; LOD = 0.21 µg/mL, LOQ = 0.71 µg/mL, peaks 4, 5, 6, and 7). ANOVA analysis—in each column, different letters indicate significant differences (*p* < 0.05).

**Table 13:** Antioxidant activity at the optimal conditions for each extract (µg/mL).

Sample	TBARS (IC_50_^1^)	OxHLIA (IC_50_^1^) Δt = 60 min
P1	850 ± 40 ^c^	61 ± 1 ^b^
P2	1600 ± 88 ^b^	62 ± 1 ^b^
P3	2510 ± 147 ^a^	540 ± 15 ^a^
Trolox *	139 ± 5 ^d^	21.8 ± 0.2 ^c^

^1^ IC_50_: extract concentration that inhibited oxidation by 50%. * Positive control. One-way analysis of variance (ANOVA)—in each column, different letters indicate significant differences (*p* < 0.05).

**Table 14:** Antimicrobial activity under the optimal conditions for each extract (mg/mL).

	P1		P2		P3		Streptomycin *		Methicilin *		Ampicillin *		Ketoconazole *	
	MIC	MBC/MFC	MIC	MBC/MFC	MIC	MBC/MFC	MIC	MBC	MIC	MBC	MIC	MBC	MIC	MFC
*Enterobacter cloacae*	10	>10	10	>10	>10	>10	0.007	0.007	n.t.	n.t	0.15	0.15	n.t.	n.t.
*Escherichia coli*	5	>10	5	>10	>10	>10	0.01	0.01	n.t.	n.t.	0.15	0.15	n.t.	n.t.
*Pseudomonas aeruginosa*	>10	>10	10	>10	10	>10	0.06	0.06	n.t.	n.t.	0.63	0.63	n.t.	n.t.
*Salmonella enterica*	5	>10	2.5	>10	>10	>10	0.007	0.007	n.t.	n.t.	0.15	0.15	n.t.	n.t.
*Yersinia enterocolitica*	10	>10	5	>10	2.5	>10	0.007	0.007	n.t.	n.t.	0.15	0.15	n.t.	n.t.
*Bacillus cereus*	>10	>10	>10	>10	>10	>10	0.007	0.007	n.t.	n.t.	n.t.	n.t.	n.t.	n.t.
*Listeria monocytogenes*	10	>10	5	>10	5	>10	0.007	0.007	n.t.	n.t.	0.15	0.15	n.t.	n.t.
*Staphylococcus aureus*	10	>10	10	>10	10	>10	0.007	0.007	0.007	0.007	0.15	0.15	n.t.	n.t.
*Aspergillus brasiliensis*	10	>10	10	>10	10	>10	n.t.	n.t.	n.t.	n.t.	n.t.	n.t.	0.06	0.125
*Aspergillus fumigatus*	>10	>10	>10	>10	>10	>10	n.t.	n.t.	n.t.	n.t.	n.t.	n.t.	0.5	1

MIC: minimum inhibitory concentration (mg/mL); MBC: minimum bactericidal concentration (mg/mL); MFC: minimum fungicidal concentration (mg/mL); n.t.: not tested. * Positive control.

## Data Availability

Not applicable.

## Sample Availability

Samples of pumpkin peels are available from the authors.
